# *Mycoplasma pneumoniae* Infections: Pathogenesis and Vaccine Development

**DOI:** 10.3390/pathogens10020119

**Published:** 2021-01-25

**Authors:** Zhulin Jiang, Shuihong Li, Cuiming Zhu, Runjie Zhou, Polly H. M. Leung

**Affiliations:** 1 Institution of Pathogenic Biology, Hengyang Medical College, University of South China, Hengyang 421001, China; jiang_zl2020@126.com (Z.J.); zhourunjie6660@163.com (R.Z.); 2 Department of Health Technology and Informatics, The Hong Kong Polytechnic University, Hong Kong 999077, China; polly.hm.leung@polyu.edu.hk

**Keywords:** *Mycoplasma pneumonia*, virulence factors, pathogenesis, whole-cell vaccine, subunit vaccines, DNA vaccines, live vector vaccines

## Abstract

*Mycoplasma pneumoniae* is a major causative agent of community-acquired pneumonia which can lead to both acute upper and lower respiratory tract inflammation, and extrapulmonary syndromes. Refractory pneumonia caused by *M. pneumonia* can be life-threatening, especially in infants and the elderly. Here, based on a comprehensive review of the scientific literature related to the respective area, we summarize the virulence factors of *M. pneumoniae* and the major pathogenic mechanisms mediated by the pathogen: adhesion to host cells, direct cytotoxicity against host cells, inflammatory response-induced immune injury, and immune evasion. The increasing rate of macrolide-resistant strains and the harmful side effects of other sensitive antibiotics (e.g., respiratory quinolones and tetracyclines) in young children make it difficult to treat, and increase the health risk or re-infections. Hence, there is an urgent need for development of an effective vaccine to prevent *M. pneumoniae* infections in children. Various types of *M. pneumoniae* vaccines have been reported, including whole-cell vaccines (inactivated and live-attenuated vaccines), subunit vaccines (involving *M. pneumoniae* protein P1, protein P30, protein P116 and CARDS toxin) and DNA vaccines. This narrative review summarizes the key pathogenic mechanisms underlying *M. pneumoniae* infection and highlights the relevant vaccines that have been developed and their reported effectiveness.

## 1. Introduction

Community-acquired pneumonia (CAP) is associated with high morbidity and mortality, and the disease is also a major threat to public health worldwide [1]. About 8–40% of CAP in children admitted to hospitals were caused by *Mycoplasma pneumoniae* [2,3,4]. Based on the reported cases in China, *M. pneumoniae* infections accounted for 19.2% of all CAP cases in adults, and the prevalence of CAP in children and teenagers, ranged from 10% to 30% [1,5]. In the USA, a recent study of 2254 hospitalized children with CAP showed that 8% children with median age of 7 years were positive for *M. pneumoniae* by polymerase chain reaction (PCR) [6].

Airborne droplets containing *M. pneumoniae* can be transmitted and spread among people through coughing and sneezing. *M. pneumoniae* causes both upper and lower respiratory tract infections, and in most cases the clinical symptoms are non-specific [7]. Tracheobronchitis is the most common type of lower respiratory infection, the incidence of which is about 20 times that of pneumonia, and 10–40% of respiratory tract infections caused by *M. pneumoniae* will eventually develop into pneumonia [8]. While most pneumonia caused by *M. pneumoniae* (MPP) cases are benign, some cases may develop into severe pneumonia and refractory pneumonia with pleural effusion, multi-organ dysfunction, and serious long-term sequelae, including bronchiolitis obliterans and bronchiectasis [9]. Although CAP is the most significant disease caused by *M. pneumoniae*, the pathogen is known to cause upper respiratory tract infections. Pharyngitis is commonly reported while rhinosinusitis and otitis media are less frequently encountered in upper respiratory tract infections caused by *M. pneumoniae* [7].

*M. pneumoniae* respiratory infections are associated with asthma exacerbation during which patients will suffer from a combination of symptoms including sudden or progressive coughing, respiratory distress, wheezing or chest pain [10,11]. The onset of asthma is due to the release of Mycoplasma-mediated cytokine in infected patients [12]. Respiratory infections caused by *M. pneumoniae* are also associated with a wide array of extrapulmonary manifestations such as meningoencephalitis, myocarditis, nephritis, atherosclerosis and mucocutaneous eruptions, etc. [13,14,15,16,17]. More importantly, *M. pneumoniae* induces mucocutaneous diseases include Stevens-Johnson syndrome and *M. pneumoniae*-associated mucositis. These mucocutaneous diseases are frequently associated with systemic inflammation and higher risk of the occurrence of long-term sequelae [18,19,20,21].

Due to the atypical symptoms produced during *M. pneumoniae* infection, pneumonia can be underestimated during the early stage of infection. There are no distinctive clinical or radiographic features in patients with *M. pneumoniae* infections, so laboratory diagnosis mainly based on rapid culture of throat swab specimens, PCR and serological assays. Furthermore, enzyme-linked immunosorbent assays (ELISA) detecting the *N*-terminal fragment of P116 protein and the C-terminal region of P1 protein both hold promise for serodiagnosis [22,23]. The IgM ELISA assays based on the short recombinant P116 and P1 proteins were shown to improve the specificity of the immunodiagnostic assay [22].

Although *M. pneumoniae* infection is generally self-limiting and does not require antibiotic treatment, patients of all age groups can develop severe, life-threatening or extrapulmonary diseases [24]. Antibiotics such as tetracycline and fluoroquinolone have been reported to be effective in eliminating *M. pneumoniae* infections [25] but tetracyclines cause discoloration of bones and teeth in young children. Fluoroquinolones can also affect the muscle, joint and tendon. Instead, macrolides, which have fewer side effects, have been the drug of choice for treating *M. pneumoniae* infection in past years [26]. More worrisome is that the extensive use of macrolides in China has led to a particularly high rate of macrolide resistance in this organism (69%~95%) [27]. The emergence of antibiotic resistance represents another challenge regarding the treatment of *M. pneumoniae* infections. Failure in antibiotic treatment has caused an increase in mortality rate during recent years [28]. Although the clinical outcomes of infections caused by macrolide-susceptible and -resistant *M. pneumoniae* isolates are not significantly different, patients infected with macrolide-resistant isolates had a longer febrile period (1.71 days), length of hospital stay (1.61 day), antibiotic drug courses (2.93 days), and defervescence time after macrolide treatment (2.04 days) compared to patients infected with macrolide-sensitive isolates [29]. Furthermore, macrolide-resistant strains may be associated with more extrapulmonary complications, and severe clinical and radiological features [24,30]. Hence, the development of vaccines against *M. pneumoniae* infections is a potential solution for the prevention of infections caused by the pathogen.

## 2. Virulence and Pathogenesis of *M. pneumoniae*

*M. pneumoniae* encodes a variety of virulence factors, which include adhesins, glycolipids, toxic metabolites, community-acquired respiratory distress syndrome (CARDS) toxin, and capsular polysaccharides. Table 1 summarizes the key virulence factors associated with *M. pneumoniae.*

### 2.1. Adhesins

**Figure 1:**
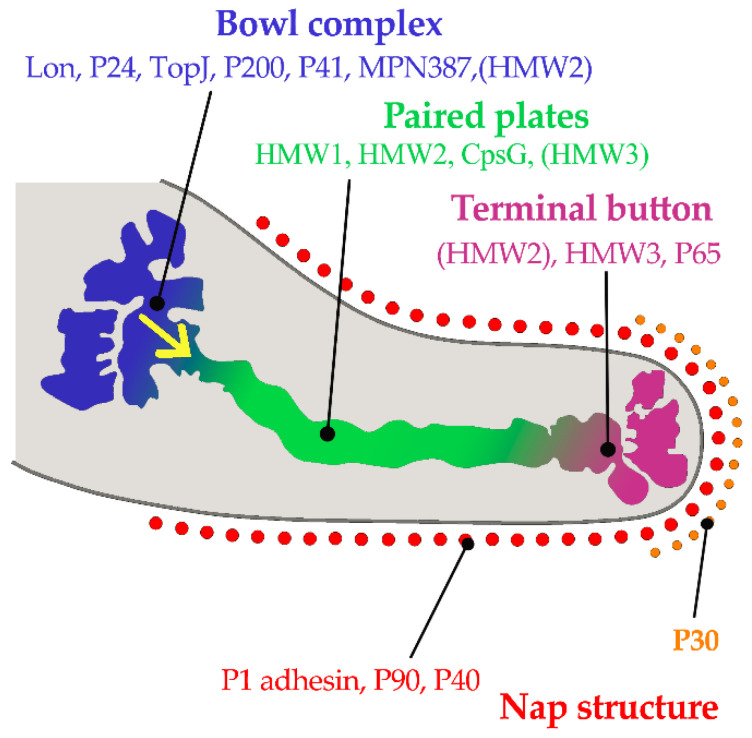
Component proteins of the internal structure of attachment organelle and proposed mechanism of movements for gliding in *M. pneumoniae*. HMW1, HMW2, and HMW3 refer to three high molecular weight (HMW) proteins. The force is generated at the bowl complexes, transmitted through the paired plates, and reaches the P1 adhesin complex in the direction of the yellow arrow. (Based on ideas from Nakane, et al. [58]). Copyright: ©2015. Public Library of Science. Creative Commons Attribution License and disclaimer available from: http://creativecommons.org/licenses/by/4.0/.

*M. pneumoniae* attaches to epithelial cell surfaces with a high affinity for human respiratory epithelial cells. The pathogen has no cell wall and colonizes the respiratory tract via its specific attachment organelle, which is a protrusion at one end of the *Mycoplasma pneumoniae* cell (Figure 1). The attachment organelle consists of internal and surface structures [58]. The internal structure is made up of a dumbbell-shaped terminal button consisting of three protein molecules (HMW2, HMW3, and P65), paired plates (HMW1, HMW2, CpsG, and HMW3), and a bowl complex (Lon, P24, TopJ, P200, P41, MPN387, and HMW2). The Nap structure in the surface adhesion complex consists of the main adhesins (P1 and P30) and accessory proteins (P40 and P90) surrounding the cell membrane (Figure 1). During gliding, the force generated at the bowl complexes is transmitted through the paired plates and reaches the P1 adhesin complex [58]. P30 adhesin is a membrane protein at the distal end of the attachment organelle, required for cytoadherence, gliding motility and stabilization of the accessory protein P65 [33]. Interaction of the *M. pneumoniae* attachment organelle with the host’s respiratory epithelium induces cytoskeleton rearrangement in the host cell, which promotes intracellular delivery of the pathogen [59,60].

The host receptors for *M. pneumoniae* are sialylated glycoproteins on the respiratory epithelium. The nature and density of host receptor moieties affect the attachment and gliding mobility of the pathogen. P1 adhesin binds to both α-2,3 and α-2,6 linkages, but only the latter type of linkage supports gliding of *M. pneumoniae* [61].

Attachment and invasion of *M. pneumoniae* produces direct damage to the host’s respiratory epithelium [59,62]. Disturbance of carbohydrate metabolism, amino acid intake and protein synthesis of the host cell results in nutrient depletion [46] Furthermore, the oxygen radicals generated by the pathogen in the host cell can lead to cilia destruction and host cell damage [63,64].

### 2.2. Inflammation Injury

Bacterial cellular components, metabolites and toxins released from *M. pneumoniae* are able to induce damage in the host tissues. These include cytotoxicity, oxidative damage, apoptosis and immune-pathological damage.

#### 2.2.1. Enzymes and Metabolites

The enzyme, HapE, of *M. pneumoniae* is a virulence factor that can produce H_2_S by the desulfurization of cysteine [47,48] which can lead to erythrocyte lysis. This enzyme mediates inflammatory reactions via adenosine triphosphate (ATP)-sensitive K^+^ channels [65]. Oxidation of glycerol by the pathogen produces toxic metabolites [66] including hydrogen peroxide [67,68] which injures cells by causing inflammation. In addition, the Ca^2+^-dependent cytotoxic nuclease (encoded by MPN133) produced by *M. pneumoniae* can lead to apoptotic-like programmed cell death in the host.

#### 2.2.2. Lipoproteins

More than 50 different lipoproteins have been identified in *M. pneumoniae*, many of them involved in inflammatory reactions [69]. The transcription of *M. pneumoniae* lipoprotein genes are regulated in response to changes in environmental conditions (e.g., oxidative and acidic stress) [70,71]. The *N*-terminal region of all the lipoproteins contains a lipid-cysteine structure and these lipoproteins induce inflammation [69]. *M. pneumoniae* lipoproteins can be recognized by toll-like receptor (TLR)1, TLR2 and TLR6, which stimulate the release of proinflammatory cytokines including tumor necrosis factor (TNF)-α, interleukin (IL)-1β, IL-6 and other inflammatory mediators via the nuclear factor κB (NF-κB) pathway [72,73].

#### 2.2.3. Community-Acquired Respiratory Distress Syndrome (CARDS) Toxin

The CARDS toxin encoded by *MPN*372 is a unique bacterial adenosine diphosphate (ADP)-ribosylating and vacuolating toxin produced by *M. pneumoniae* [74,75]. The structure of CARDS toxin comprises a triangular molecule in which *N*-terminal mono-ADP ribosyl-transferase (mART) and C-terminal tandem β-trefoil domains associate to form a unique overall architecture different from other well-recognized ADP-ribosylating bacterial toxins [53]. CARDS toxin demonstrates high binding affinity to human surfactant protein A and annexin A2 when present in the airway epithelia and exhibits specific biological activities including mono-ADP ribosylation and vacuolization [53,74]. CARDS toxin binds to mammalian cell surface receptors and is internalized rapidly in a dose and time-dependent manner. The internalization process is mediated by clathrin molecules, which form a molecular scaffold for uptake of CARDS toxin [76]. The toxin is cytotoxic to mammalian cells by activation of the NLRP3-associated inflammasome and further promotes the release of IL-1β and IL-18 [77,78,79]. CARDS toxin increases the expression of the proinflammatory cytokines IL-1β, IL-6 and TNF-α in a dose- and activity-dependent manner [80]. CARDS toxin is capable of inducing an allergic-type inflammation in animals [81,82], but there is no convincing evidence that CARDS toxin is a causal factor of *M. pneumoniae*-associated asthma.

#### 2.2.4. Lipids

The cell membrane of *M. pneumoniae* has a high lipid content (comprising primarily of the acidic glycerophospholipids phospholipids and cholesterol), which can infiltrate the host epithelial cells, disrupt the lipid bilayer of the cell membrane and cause leakage of ionic metabolites [54,55]. Furthermore, some scholars speculate that these lipids may act as potential TLR4 ligands for binding to TLR4 and elicit macrophage autophagy, eventually leading to the secretion of proinflammatory cytokines [50,83] and triggering typical host cell inflammatory responses [50,84].

#### 2.2.5. Capsules

*M. pneumoniae* has a capsular structure made up of polysaccharides [52] which may be potential virulence factors and are immunogenic, but its functional role in pathogenesis remains unclear and needs to be further explored [85,86,87].

### 2.3. Immune Evasion

*M. pneumoniae* has multiple strategies to escape host immune responses in order to ensure survival of the pathogen. Its survival includes immune evasion which may play an important role in pathogenesis. Inadequate immune responses against the invading pathogen results in uncontrolled proliferation and host tissue damage [88].

#### 2.3.1. Molecular Mimicry

The term molecular mimicry can be described simply as “pathogens sharing a structural relationship with the host are tolerated as self, just like constituents of the host” [2,88,89]. The immune response targets the pathogen-peptide mimicking the host’s self-antigen, leading to the activation of naive, autoreactive T-cells specific to the corresponding self-antigen [89]. *M. pneumoniae* antigen mimics host cell components, thus the host immune response induced by the pathogen causes auto-immune responses and injuries to multiple organs [2,90].

The C-terminal region of the P1 and P30 proteins in *M. pneumoniae* show high levels of homology to troponin, cytoskeletal proteins, keratin and fibrinogen of the host [46,91]. Antibodies produced in response to *M. pneumoniae* infections will target various host tissues and form immune complexes, which aggravates the autoimmune response, leading to inflammatory injuries in the extrapulmonary tissues [13,46].

#### 2.3.2. IbpM

Immunoglobin binding protein (IbpM) is a surface protein encoded by *MPN400* that binds strongly to various immunoglobulins (IgM, IgG, and IgA) produced by the host [45]. Blötz et al. demonstrated that IbpM was required by *M. pneumoniae* to produce cytotoxic effects in host cells and is thus regarded as a virulent factor [45].

#### 2.3.3. Antigen Variation

It has been observed that the surface adhesins P1, P40, and P90 of *Mycoplasma pneumoniae* display sequence variation [92,93]. Sluijter et al. demonstrated that the RecA protein homolog encoded by MPN490 promoted gene exchange between homologous DNA sequences (RepMP) in *M. pneumoniae* [94]. The RepMP are repetitive sequences present within genes encoding surface proteins such as the adhesins. Homologous recombination between these RepMP sequences generates sequence changes within the adhesin genes, which results in variations of surface adhesins and facilitates evasion of host immune surveillance [94,95,96].

The role of post-translational modifications of *M. pneumoniae*-specific proteins (e.g., P1, P40, P90) is a relatively new aspect of bacterial epigenetics [34]. The posttranslational modification of cytoadherence proteins by the protein kinase PrkC is essential for the development and function of the *M. pneumoniae* terminal organelle [97]. P1 adhesin of *M. pneumoniae* M129 is subject to extensive post-translational processing forming 22 proteo-forms, which are specific molecular forms of a protein product arising from a specific gene. Each of the proteo-forms retain the ability to bind to host molecules or their structural mimics and are surface accessible [31]. There are many issues that require further study, such as whether the antigen variations caused by post-translational modifications can affect the pathogenicity of *M. pneumoniae*.

#### 2.3.4. Intracellular Survival

*M. pneumoniae* can survive for a long time in the human lung carcinoma cell (A549) [98], but the pathways related to intracellular survival remain to be elucidated. Intracellular *M. pneumoniae* has mechanisms to protect the pathogen against phagocytosis and antibiotics. This may explain why *M. pneumoniae* infection can develop into chronic lung disease, such as refractory pneumonia caused by macrolide-resistant *M. pneumoniae* due to the lack of timely and effective antibiotic treatment.

#### 2.3.5. Others

Moreover, *M. pneumoniae* has an antioxidant mechanism to protect against oxidative reactions such as reactive oxygen species (ROS) damage [46,99]; A nuclease encoded by *MPN49*1 can degrade neutrophil extracellular traps (NETs), which helps the pathogen to escape from the immune attack of host cells [44].

**Figure 2:**
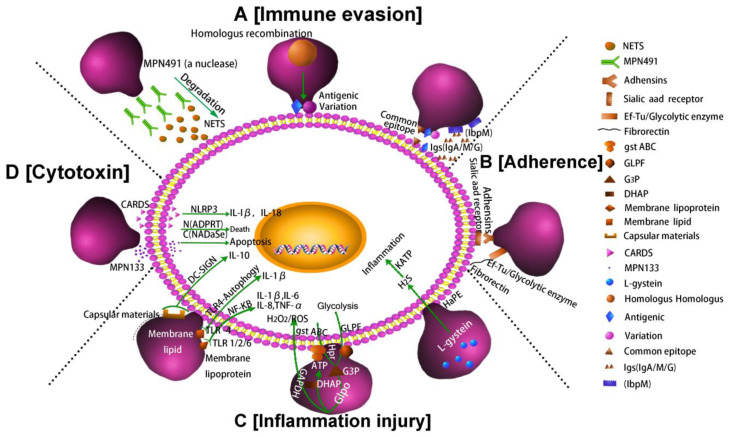
Pathogenic mechanisms of *M. pneumoniae*. (**A**) Nuclease and IbpM in *M. pneumoniae* enable immune evasion, and homologous DNA recombination leads to antigen variation; (**B**) *M. pneumoniae* adhesion causes cell damage. Additionally, the P1 adhesin protein binds to the sialic acid receptor on the host cell surface contributing to *M. pneumoniae* adherence and gliding. Furthermore, elongation factor Tu (EF-Tu) can bind strongly to a diverse range of host molecules (such as fibronectin), contributing to adhesion; (**C**) Inflammation-inducing factors (HapE enzyme, oxidase GlpO, membrane lipids, lipoproteins, and capsular materials) activate host cell inflammatory pathways; (**D**) *M. pneumoniae* secretes cytotoxic nuclease (catalytic protein encoded by *MPN*1*33*) and CARDS toxin.

In summary, the pathogenesis of *M. pneumoniae* involves mainly the following four factors: immune evasion, adhesion, inflammatory injury and cytotoxicity. Figure 2 shows these four key pathogenic mechanisms of *M. pneumoniae* infection.

## 3. Development of Vaccines against *M. pneumoniae* Infections

*M. pneumoniae* is a significant bacterial pathogen causing CAP. The lack of cell wall in *M. pneumoniae* greatly reduces the choice of current antibiotics. Furthermore, the increased number of refractory infections caused by macrolide-resistant *M. pneumoniae* makes clinical treatment extremely difficult, especially for children. Although the mortality and disability rates caused by *M. pneumoniae* infection are low, complications, and even fatal pneumonia, can occur in susceptible individuals (children of 5–15 years, adolescents, and the elderly of >60 years) in an epidemic area. At present, no vaccine is available for protection against *M. pneumoniae* infections. In view of the increasing importance of *M. pneumoniae* infection, there is an urgent need for an effective vaccine.

The types of vaccines that are most studied in *M. pneumoniae* include inactivated, live-attenuated, protein subunit and recombinant DNA vaccines. The vaccines are mainly developed to be administered via the nasal or parental route. It has been reported that nasal administration of inactivated vaccine elicited low levels of protection resulting in a high reinfection rate [100]. For vaccinated individuals who did not produce protective antibodies, *M. pneumoniae* reinfection could lead to an early hyper-accentuated histopathological response [100,101].

### 3.1. Whole-Cell Vaccines

Various *M. pneumoniae* vaccines developed from whole-cell antigen, including both inactivated and live-attenuated vaccines, have been reported since 1964. The protective effects of these vaccines were tested on military personnel [102,103,104,105,106], a small number of volunteers [107,108,109,110] and animal models [111,112]. These vaccines have been reported to have low efficiencies in reducing the incidence and disease severity [113,114,115]. A meta-analysis suggested that inactivated vaccines reduced the incidence of both *M. pneumoniae* (MPP) and respiratory infections by ~40% only [100]. Inactivated *M. pneumoniae* vaccines supplemented with alum adjuvant and increased dosage of vaccines were found to improve the immunogenicity and protective efficiency [116]. However, the improvement in vaccine efficiencies was observed in animal models, but still less effective in humans [113,114,115]. After inoculating with inactivated vaccines, most of recruited subjects showed no significant adverse reactions (including autoimmune responses) and vaccination did not exacerbate disease upon subsequent homologous challenge [117,118,119]. However, disease was more severe in human subjects who lacked an antibody response following vaccination [110].

In comparison, there were less studies of live-attenuated vaccines against *M. pneumoniae*. Live-attenuated vaccines were often prepared by continuous passage in vitro [107,111,120] and/or produced by temperature-sensitive mutants [121,122]. Live-attenuated vaccines were found to induce protective effect in hamsters. Clinical trials for efficiency evaluation of live-attenuated vaccine were never performed in humans because of the significant health risk involved [107,109,110].

### 3.2. Recombinant Protein Subunit Vaccines

As a group of important adhesion factors in *M. pneumoniae*, P1, P30 and other adhesion-associated protein have immunogenicity and immunoreactivity and are able to induce specific neutralizing antibodies. Currently, the strategies involved in the preparation of recombinant protein subunit vaccines include the employment of cell or cell-free protein synthesis system. So far, a number antigen targets for vaccine development were identified (Table 1). Among these virulence factors, cytoadherence proteins (including P1, P30, P116, CARDS toxin), polysaccharides, lipids and lipoproteins have immunogenicity and are likely to be potential candidates for vaccines antigens.

#### 3.2.1. P1 Adhesin Protein Vaccine

P1 plays an important role in the pathogenesis of *M. pneumoniae* infection by mediating the attachment of the pathogen to host cells [123], and the *p*1 gene is used as target to detect *M. pneumoniae* by qRT-PCR, as well as to perform genotyping [124,125]. Although it is unclear whether genotype-specific antibodies have an influence on re-infections due to different genotypes of *M. pneumoniae*, genotyping is also crucial for the molecular epidemiological studies and the development of an effective vaccine [126]. Protein P1 is a transmembrane adhesin, and it has high immunogenicity and antigenic specificity [127,128] which means its epitopes were not or rarely found in other bacterial species. Intramuscular or intranasal inoculation of BALB/c mice with a DNA vaccine encoding amino acid 1125–1359 of the *M. pneumoniae* P1 protein C-terminal region (P1C) led to detectable protection against *M. pneumoniae* infection. The levels of IgG (IgG1, IgG2a, and IgG2b isotypes) and cytokines (IFN-γ and IL-4) were significantly elevated [129]. However, the effect of P1C DNA vaccine in humans remains unknown and requires further research.

#### 3.2.2. P30 Vaccine

P30 is a transmembrane protein and is required for host receptor binding [58]. Similar to P1, P30 is also an important immunogenic factor [130]. Mutant *M. pneumoniae* without the gene encoding P30 is noninfectious and unable to adhere to host cells [45], which suggests that P30 could potentially be an ideal candidate target for a clinical vaccine. Szczepanek et al. created an avirulent P30 adhesin mutant for assessing its efficacy as a live-attenuated vaccine candidate in mice [112]. However, the live-attenuated vaccine caused severe complications in BALB/c mice, which appears to be driven by responses of the T helper type 17 (Th17) cells. In the recent years, a large number of studies have reported that Th17 cells play an important role in antimicrobial immune responses and causing autoimmune diseases in mouse models [131,132].

On the other hand, vaccine produced from recombinant P30 adhesin was found to elicit immune protection. Hausner et al. (2013) created a recombinant protein by combining protein P30 (amino acids 17 to 274) with the C-terminal of P1 adhesin (amino acids 1287–1518 of P1) [133]. When this recombinant vaccine was injected into guinea pigs, protective IgA were secreted in the respiratory tracts of the animals. These results provide insights into vaccine development for effective protection against *M. pneumoniae* infection in humans.

#### 3.2.3. P116 Vaccine

P116 protein is another major antigen of *M. pneumoniae* and an important cellular adhesion factor [36,134]. It is a 116kDA protein consisting of 1030 amino acids. Svenstrup et al. has purified the P116 protein and found that polyclonal antibodies raised against this protein prevented *M. pneumoniae* adhesion to Hep-2 cells [135]. Additionally, the serum obtained from *M. pneumoniae*-infected patients contained antibodies that specifically reacted with P116 [136].

#### 3.2.4. CARDS Toxin Vaccine

As mentioned elsewhere in this review, CARDS toxin is a specific virulence factor associated with *M. pneumoniae* pathogenesis. The amount of toxin produced is positively correlated with the severity of pulmonary disease [46,76]. In a BALB/cJ mouse model, CARD toxin dosage was correlated with inflammatory responses characterized by airway restriction and decreases in lung compliance [81]. Antibody against CARDS toxin was identified in the serum of *M. pneumoniae*-infected patients during both the acute infection and recovery periods, with higher levels in the recovery period. Analysis of serum antibodies on day 28 after the onset of *M. pneumoniae* infection showed that antibody against CARDS toxin was positive, while low levels of CARDS toxin-reactive antibodies were identified in the serum of healthy controls [75]. There was study showing that, the C-terminal region of CARDS toxin triggered an antibody response upon *M. pneumoniae* infection [137], this provides insights into the development of vaccine using attenuated CARDS toxin.

#### 3.2.5. Recombinant Combined Vaccines

As described earlier, some apical organelle-localized proteins (P1, P30, P116, etc.) had immunogenicity [22,130]. Hence genetically engineered recombinant proteins by screening antigen dominant epitopes to stimulate the humoral immune response are promising vaccine candidates for preventing *M. pneumoniae* infection. Chen et al. designed a chimeric protein (P116N-P1C-P30), designated MP559, which contained various antigen epitopes of three antigens [138]. Vaccination with MP559 stimulated the same humoral immune response as the three antigens alone. The study showed that chimeric protein MP559 has the potential to replace the three individual protein subunit vaccine candidates.

#### 3.2.6. Other Vaccines

In addition, the success of capsular polysaccharide vaccines against *Streptococcus pneumoniae* and *Neisseria meningitidis* provides enlightenment for the use of purified specific polysaccharides to develop a *M. pneumoniae* vaccine. But a recent study has revealed that antibodies to protein but not glycolipid structures are important for host defense against *M. pneumoniae* [139]. So, vaccines using other virulence factors as antigens (such as polysaccharides, lipids, glycolipid) may be unable to induce persistent protective immunity and the molecular mimicry-induced cross-reactive immunity may cause injury to multiple organs.

### 3.3. DNA Vaccines

DNA vaccines are emerging biotechnology products that involve novel approach to induce immune responses against the target immunogens. The immunogen is expressed in vivo from a DNA vector carrying the gene encoding the immunogen [140]. Compared with traditional vaccines, DNA vaccines have certain obvious advantages. First, they can be easily constructed and pose no infection risk. Second, they trigger immune responses corresponding to those produced against natural antigens. Third, the cost of producing, storing, and transporting DNA vaccines is lower than the cost associated with protein vaccines. DNA vaccines for preventing *M. pneumoniae* infection was shown induce both strong humoral and cell-mediated immunity (Th1 and Th2 responses), although antibody production by B-cells has been shown to be lower than that associated with traditional vaccines [141,142,143]. In addition to the *P*1*C* DNA vaccine [129], another DNA vaccine produced by fusing *P*1*C* with the *E. coli* heat-labile toxin B subunit (*LTB*) gene has been studied [144]. The *LTB-P*1*C* fusion DNA vaccine was shown to stimulate immune protection against *M. pneumoniae* infection in a BALB/c mouse model, with less pathological inflammation [144]. Besides, the study also demonstrated that production of *M. pneumoniae*-specific IgA and IgG2a/IgG1 ratios in the bronchoalveolar lavage fluid and sera were significantly higher in mice vaccinated with the *LTB-P*1*C* fusion DNA vaccine than in mice vaccinated with the *P*1*C* DNA vaccine [144]. Of course, there are also multiple obstacles that need to be overcome before DNA vaccines can be used in humans. These hurdles include target gene selection, fate of the injected DNA, immune tolerance, potential integration of the injected DNA material with human chromosomes and uncontrolled expression in vivo.

### 3.4. Live Vector Vaccines

In 1980, a Swiss scholar first reported DNA (SV40 DNA) can be transferred from bacteria to higher organisms (e.g., CV-1 cells), which also laid the foundation for research of live vector vaccines (LVVs) [145]. LVVs are produced by introducing a specific antigen gene into known bacteria using plasmid vector or by integrating into the bacterial chromosome. When the live bacteria are being taken orally by the vaccine recipient, the antigen gene is being expressed by the bacteria inside the recipient’s body to produce an antigen and then stimulate a specific immune response. The bacterial strains used in LVVs are avirulent organism (e.g., *Bacillus subitilis*), probiotics (*Lactobacillus*) or attenuated strains (e.g., attenuated *Listeria monocytogenes* strains) [146]. LVVs are associated with several advantages, including safety, stimulation of long-term humoral and mucosal immunity, and multivalent vaccines, making LVV promising candidates for successful vaccination. There are also certain issues, including unstable expression of foreign genes and reversion of the live-attenuated vector to a virulent form.

In the recent years, there are more LVVs that have been studied and some of them exhibited the potential for infection prevention, which makes the idea of developing an LVV to prevent *M. pneumoniae* infection more promising. Currently, there is no LVVs for preventing *M. pneumoniae* infection, but there is one for preventing infection of pigs with *Mycoplasma hyopneumoniae* [147]. Vectors for constructing LVVs mainly involve symbiotic bacteria, probiotics (e.g., *Lactobacillus* spp.), normally harmless bacteria and attenuated microorganisms [148,149,150,151]. Examples of harmless bacteria include *Bacillus subtilis* [152,153] and *Saccharomycetes* [154,155]. Examples of live-attenuated microorganisms that have been widely used as live vaccine vehicles include *Salmonella* [156,157], *Listeria* [158,159], poxvirus [160] and influenza virus [161]. Li et al. constructed a recombinant *Lactobacillus*-derived vaccine that displayed influenza epitopes (sM2 and HA2) [162]. Ferreira et al. also explored the immune efficiency of a recombinant *Lactobacillus casei*-derived vaccine that expressed a fusion protein involving pneumococcal PspA and PspC [163]. Based on the findings of these studies, LVV is a promising strategy for development of multiple epitopes-vaccine for *M. pneumoniae* using probiotics as vehicles.

## 4. Summary and Future Prospects

*M. pneumoniae* is the most common pathogen leading to atypical CAP, occasionally with extrapulmonary manifestations. Worse still, chronic refractory MPP can lead to serious complications. Refractory *M. pneumoniae* infections caused by macrolide-resistant *M. pneumoniae* have become more common in China, especially in children, which makes treatment more difficult. Owing to these reasons, there is an urgent need for development of effective vaccines for preventing *M. pneumoniae* infections.

There are various types of *M. pneumoniae* vaccines including inactivated, live-attenuated, and subunit vaccines. These vaccines are mainly administered via the nasal route or hypodermic needle. Whole-cell vaccines can be either inactivated or live-attenuated vaccines. Inactivated vaccines were found to elicit weak immune responses, and some individuals who did not produce antibodies after vaccination experienced severe immune responses on reinfection with *M. pneumoniae*. Vaccines based on *M. pneumoniae* adhesion proteins (P1, P30 and P116) have been considered as promising options. Hypodermic inoculation with a protein subunit vaccine results in effective immune protection [164]. However, the generation of a protein subunit vaccine can be challenging because they are accompanied by several inevitable shortcomings and technical difficulties. (1) The protein expression level in vitro is usually very low. Besides, purification of recombinant proteins is a complicated process [165,166]. (2) Protein subunit vaccines do not have a self-replicating ability compared with live attenuated vaccines or DNA vaccines, so multiple immunization is usually required [167]; (3) The protein subunit may lose its natural conformation when being expressed in heterologous systems [168]. DNA vaccines trigger both cell-mediated and humoral immunity, but the injected DNA cannot be consistently replicated in mammalian cells. These challenges need to be overcome in the future to develop effective vaccines.

*M. pneumoniae* infections in immunocompetent patients induce antibody responses that mainly direct against the terminal organelle-associated proteins in *M. pneumoniae* [169]. Thus, vaccine based on this adhesin can induce specific immunoglobulins that inhibit the adherence of *M. pneumoniae* to the respiratory epithelium of the host [169]. However, due to their weak humoral immunogenicity when used alone without aluminum adjuvant, a fusion protein with an adjuvant, such as hepatitis B virus capsid HN-144 fragment, is a preferred immunization strategy. Although LVVs using probiotics as expression vectors are still in its exploratory stage, it is believed that LVV could be a promising vaccine strategy against *M. pneumoniae* infections in the near future. The use of living probiotics as expression vector of LVVs enables vaccine delivery through pulmonary atomization and/or oral ingestion. We regard protein vaccines (usually manufactured by means of genetic engineering) are the most promising vaccines for the prevention of *M. pneumoniae* infections. Single-antigen protein formulated with vaccine adjuvant and multi-epitope fusion protein are promising vaccine candidates. Regardless of the vaccine type, the immunogenicity, safety, effectiveness, and functional mechanisms of vaccines used in humans need to be thoroughly researched before further clinical trials can be commenced.

At present, *Coronavirus* disease 2019 (COVID-19) has spread to almost every part of the world. COVID-19 co-infection with other common respiratory pathogens such as *M. pneumoniae* is not unexpected [170,171]. COVID-19 co-infection with *M. pneumoniae* may exacerbate clinical symptoms, delay recovery time, and increase morbidity and mortality [172,173], while vaccines for COVID-19 is on the way. There is no knowledge so far about whether co-infection of COVID-19 and *M. pneumoniae* will affect the outcome of vaccination. Although viral infection and bacterial infection are essentially different in terms of pathogenesis, some clinical manifestations of COVID-19 are similar to MPP, such as fever, dry cough, fatigue, ache all over, chest tightness, etc. [174,175]. Clinical practice indicates that reactive lymphocytes are frequently seen in COVID-19 infection, while in *M. pneumoniae* infection cold agglutination is common [172,174]. There are only subtle differences in radiographic features (chest X-ray and CT imaging) between these two diseases [171,174,176]. Genetic and serologic tests (e.g., serum IgM/IgG antibody rapid test) have definitely helped clinicians to diagnose and manage COVID-19 patients during the COVID-19 pandemic [173]. There are some questions as to whether COVID-19 or COVID-19 co-infection with *M. pneumoniae* will be in existence in humans for a long time. What is the mechanism via which COVID-19 co-infection with *M. pneumoniae* increases morbidity and mortality? We can speculate from our research experience: human cellular immunity may be suppressed by one pathogen with an immune escape mechanism, which causes a declining antigen reactivity to another pathogen. Is this true? Are there other mechanisms? These topics may be research hotspots in the future.

## Figures and Tables

**Table 1:** Key virulence factors of *M. pneumoniae*.

Pathogenic Mechanism	Virulence Factor	Gene Annotation	Reference
Adherence	P1	*MPN* *141*	[31]
	P30	*MPN* *453*	[32,33]
	P40 (Protein C)	*MPN* *142*	[34]
	P90 (Protein B)	*MPN* *142*	[34]
	P200	*MPN* *567*	[35]
	Hypothetical protein HMW1-3 (high molecular weight)	*MPN* *447/310/452*	[36]
	P116	*MPN* *213*	[36]
	P65	*MPN* *309*	[37]
	Elongation factor thermo unstable (EF-Tu)	*MPN* *665*	[38,39,40]
	Pyruvate dehydrogenase subunit B	*MPN*392	[41]
	Glycolytic enzymes enolase	*MPN*606	[41,42]
	TopJ	*MPN* *119*	[43]
Immune evasion	Nuclease	*MPN* *491*	[44]
	Immunoglobin binding protein (IbpM)	*MPN* *400*	[45]
Inflammation injury	H_2_O_2_	*/*	[46]
	Reactive oxygen species (ROS)	*/*	[46]
	H_2_S	*/*	[47]
	HapE enzyme	*MPN* *487*	[47,48]
	Oxidase GlpO	*MPN* *051*	[49]
	Membrane lipids	*/*	[50]
	Membrane lipoproteins	*/*	[51]
	Capsular materials	*/*	[52]
Cytotoxicity	Community-Acquired Respiratory Distress Syndrome (CARDS) toxin	*MPN* *372*	[53,54]
	Cytotoxic nuclease	*MPN* *133*	[55]
Gliding motility	P65	*MPN* *309*	[37]
	P30	*MPN* *453*	[32]
	Hypothetical protein MPN387	*MPN* *387*	[56]
	P24	*MPN* *312*	[57]
	P41	*MPN* *311*	[57]
